# Enhanced MRI *T*_2_ Relaxivity in Contrast-Probed Anchor-Free PEGylated Iron Oxide Nanoparticles

**DOI:** 10.1186/s11671-017-2084-y

**Published:** 2017-04-27

**Authors:** Bibek Thapa, Daysi Diaz-Diestra, Juan Beltran-Huarac, Brad R. Weiner, Gerardo Morell

**Affiliations:** 1 Department of Physics, University of Puerto Rico, San Juan, PR 00931 USA; 2 Molecular Sciences Research Center, University of Puerto Rico, San Juan, PR 00926 USA; 3 Institute for Functional Nanomaterials, University of Puerto Rico, San Juan, PR 00936 USA; 4 Department of Chemistry, University of Puerto Rico, San Juan, PR 00931 USA

**Keywords:** PEGylation, Magnetic iron oxide nanopaticles, *T*_2_ relaxivity, MRI contrast agents

## Abstract

**Abstract:**

Superparamagnetic iron oxide nanoparticles (SPIONs, ~11-nm cores) were PEGylated without anchoring groups and studied as efficient MRI *T*_2_ contrast agents (CAs). The ether group of PEG is efficiently and directly linked to the positively charged surface of SPIONs, and mediated through a dipole-cation covalent interaction. Anchor-free PEG-SPIONs exhibit a spin-spin relaxivity of 123 ± 6 mM^−1^s^−1^, which is higher than those of PEG-SPIONs anchored with intermediate biomolecules, iron oxide nanoworms, or Feridex. They do not induce a toxic response for Fe concentrations below 2.5 mM, as tested on four different cell lines with and without an external magnetic field. Magnetic resonance phantom imaging studies show that anchor-free PEG-SPIONs produce a significant contrast in the range of 0.1–0.4 [Fe] mM. Our findings reveal that the PEG molecules attached to the cores immobilize water molecules in large regions of ~85 nm, which would lead to blood half-life of a few tens of minutes. This piece of research represents a step forward in the development of next-generation CAs for nascent-stage cancer detection.

**Graphical Abstract:**

Contrast-probed anchor-free PEGylated iron oxide contrast agent
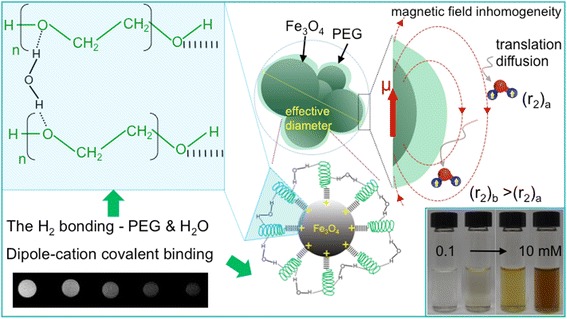

**Electronic supplementary material:**

The online version of this article (doi:10.1186/s11671-017-2084-y) contains supplementary material, which is available to authorized users.

## Background

Magnetic iron oxide nanoparticles (NPs) offer a wide scope of applications in nanomedicine, including magnetic resonance imaging (MRI), drug and gene delivery, tissue engineering, bioseparation, cell tracking and labeling, and innovative cancer therapeutics and diagnostics due to their excellent biocompatibility and unique magnetic properties [1–7]. In particular, magnetite (Fe_3_O_4_) has received the primary focus (when compared to other forms of iron oxide, such as maghemite, hematite, goethite and wustite) [8, 9] because its shape, size and surface-to-volume ratio can be synthetically modified, which facilitates larger payloads and superior stabilities useful for multiple applications in theranostics. However, the surface modification of this nanostructure using biocompatible polymers or inorganic components remains a challenge [10–18]. Greater efforts regarding the hydrophilicity concerns must be addressed in order to endow it with an enhanced colloidal stability in physiological media, low toxicity, low oxidation, and improved controlled agglomeration. Thus, superparamagnetic iron oxide NPs (SPIONs) surface-functionalized with specific biomolecules are indispensable for biomedical applications.

SPIONs are widely applied as negative probing agents in MRI, leading to the contrast in reconstructed images, which arises from the shortening of transverse relaxation time (*T*_2_) of water protons by an inhomogeneous magnetic field around the outer sphere region of NPs [19]. This relaxation time is expressed as transverse relaxation rate (*R*_2_) by the relationship,

1 $$ {R}_2=\frac{1}{T_2} = \frac{1}{T_{2,0}}+{r}_2\left[\mathrm{Fe}\right], $$

where $\frac{1}{T_{2,0}}$, *r*_2_ and [Fe] represent the transverse relaxation rate of protons in the absence of SPIONs, the transverse relaxivity of water protons, and the analytical iron concentration, respectively. In this sense, a chemical exchange reproduces nuclear magnetic relaxation caused by diffusion of water molecules near strongly magnetized SPIONs, whose transverse relaxivity can be determined by assuming that the NPs are small enough to satisfy the motional average regime (where Δω ${\uptau}_{\mathrm{D}}$ < 1, Δω and ${\uptau}_{\mathrm{D}}$ being the angular frequency shift experienced by protons and the translational diffusion time of protons in magnetic field inhomogeneity, respectively). Thus, the protons of freely diffusing water molecules that surround the NPs experience a varying dipolar magnetic field created by the electronic magnetic moments [20, 21]. Based on this outer sphere theory, the transverse relaxivity (*r*_2_) of water protons in this regime, neglecting the self-relaxation of water, is expressed as [22, 23]:

2 $$ {r}_2=\frac{R_2}{\left[\mathrm{Fe}\right]} = \left[\frac{256{\pi}^2{\gamma}^2}{405}\right].\frac{V^{*}{M_s}^2{r}^2}{\left[\mathrm{Fe}\right]\  D\ \left[1 + \frac{L}{\  r}\right]}, $$

where *γ*, *V* ^*^, *M*_s_, *r*, *D*, and *L* are the proton gyromagnetic ratio, the volume fraction of iron, the saturation magnetization, the effective radius of the magnetic NP, the diffusivity of water molecules and the thickness of impermeable surface coating, respectively. Therefore, one can predict that the higher *r*_2_ is related to the larger effective radius of the NPs, which results from the nanoparticle clustering and the formation of the impermeable polymer-coated layer. Thus, the proper choice/configuration of the biomolecules onto the magnetic cores is crucial to modify the effects of proton relaxation time reductions, and to determine the type of dipole-cation binding between the linking groups of the polymer and the positively charged surfaces of the SPIONs.

Polyethylene glycol (PEG) is one of the most common polymers used for surface functionalization of SPIONs due to its biocompatibility, hydrophilicity, nontoxicity, antifouling nature, and non-antigenicity and non-immunogenicity [24–27]. PEGs are polyether-diols with two terminal hydroxyl groups and alternating ether linkages, and are soluble in water because of the hydrogen bonding of water molecules to electron-rich oxygen atoms in the polymer chain. It has been also demonstrated that PEGylation helps avoid recognition by the reticuloendothelial system (specifically the macrophage cells), and hence extends in vivo circulation time in blood pool for both imaging and drug delivery applications [28]. In addition, PEG is inexpensive and has been approved by the US Food and Drug Administration (FDA) not only for pharmaceuticals, but also for other industries, such as food and cosmetics [29].

There have been several reports on PEGylation strategies for iron oxide based NPs [30–33]. Hu [34] and Dai [35] and their respective co-workers reported the facile PEGylation of ultrasmall iron oxide nanoparticles and SPIONs using carboxyl homobifunctionalized (-COOH-PEG-COOH) to demonstrate an efficient MRI contrast enhancement. Wang and co-workers [36] also reported a facile PEGylation process of SPIONs with PEG/polyethyleneimine (PEI) for in vivo MRI of mouse brain. Further, Tong and co-workers [37] systematically studied SPION cores coated with DSPE-mPEG copolymer, and determined that a fine tuning of the core size and impermeable PEG coating of SPIONs can further increase the *T*_2_ relaxivity per particle, which was ascribed to the fact that the PEG molecules can immobilize water molecules in a region much larger than the area of the actual iron oxide core. Similarly, Xie and co-workers [31] used dopamine as the anchoring group for an effective PEGylation of monodisperse Fe_3_O_4_ NPs through a covalent bond. They reported that the resulting NPs showed negligible aggregation for cell culture conditions, and much reduced non-specific uptake by macrophage cells [31]. In order to provide an easier and more effective method for chemical coating, Larsen and co-workers [38] reported the preparation of biocompatible iron oxide magnetic NPs coated with PEG by replacing oleic acid with a commercially available silane-anchored PEG. They found an enhanced MRI contrast in the larger coated cores, which was associated to a combined effect of the size-dependent extravasation and the capture by macrophages in certain tumors [38]. However, for such improvements, some intermediate molecules (anchoring groups) and complex PEGylation protocols are needed. There are few reports on anchor-free PEGylation of NPs [39, 40]. In addition, the feasibility of these PEGylated NPs as new-generation probing agents for MRI applications is still in its infancy. Here, we report the enhanced MRI relaxivity of 11-nm SPIONs PEGylated without anchoring groups, and its potential as an efficient *T*_2_ CA for applications in vivo.

## Methods

### Materials

All reagents used in this investigation, ferric chloride hexahydrate (FeCl_3_⋅6H_2_O, ≥99%), ferrous chloride tetrahydrate (FeCl_2_⋅4H_2_O, 99.99%), poly (ethylene) glycol (average mol. wt. 3350 Da), and ammonium hydroxide (NH_4_OH, 28.0–30.0%), were analytical-grade reagents purchased from Sigma Aldrich, USA, and were directly used without any processing.

### Synthesis of PEGylated Fe_3_O_4_

The PEG-coated magnetite NPs were synthesized via the co-precipitation method [38, 39] with minor modifications. Briefly, 1.3 mg of FeCl_3_.6H_2_O and 0.5 mg of FeCl_2_.4H_2_O were dissolved in 20 mL of deionized water, and the solution was then deoxygenated through N_2_ bubbling for 30 min, which produced a dark orange-colored solution. Afterwards, 2 mL of NH_4_OH was added dropwise under vigorous stirring in an inert environment. The solution was next heated up to 70 °C for 1 h and the flocculate was magnetically decanted several times, and then re-dispersed in deionized water obtaining a black solution of iron oxide. One third of the as-prepared solution was freeze-dried in vacuum. The resultant product was labeled as bare SPION in order to compare them to their PEG-coated counterparts. In a separate step, 40% (w/v) aqueous solution of PEG was added to two thirds of the remaining solution, being then rapidly probe-sonicated at room temperature at a frequency of 5 Hz. The resultant solution was washed many times with abundant water in order to remove uncoated PEG NPs via centrifugation at 8000 rpm for 30 min. Finally, it was freeze-dried in vacuum for 48 h obtaining powders of iron oxide labeled as PEG-SPION. The weight ratio of PEG/SPION was set at ~1:10.

### Characterization

The crystallographic phase and purity of the products were investigated with a Rigaku SmartLab X-Ray diffractometer (XRD) using CuKα (*λ* = 1.5406 Å) operating at 40 KV and 44 mA. The attenuated total reflectance (ATR) spectra of the products were obtained using a Bruker Tensor 27. The thermal behavior of SPION and PEG-SPION was studied by thermogravimetric analysis (TGA) and differential scanning calorimetry (DSC) in the temperature range of 30 to 800 °C in presence of constant N_2_ flow of 20 ml/min using PerkinElmer STA 6000 Simultaneous Thermal Analyzer. The static size, morphology, crystallinity and size distribution of the products were recorded using a JEOL JEM-2200FS high-resolution transmission electron microscope (HRTEM) operating at 200 kV. To prepare the TEM samples, 30 μL of each product in solution (0.8 mM) was dropped on lacey carbon 300 M Cu grids, and dried overnight. The hydrodynamic diameter was determined by dynamic light scattering (DLS) technique and zeta potential of bare SPIONs and PEG-SPION were determined by using a Malvern Zetasizer Nanoseries Nano-ZS (Malvern Instruments, Malvern, UK). The dispersed samples in the solvents were measured without filtration. The magnetic properties of the products were studied using a vibrating sample magnetometer (VSM, Lakeshore 7400) and a Quantum Design (PPMS DynaCool). The relaxivity measurements were performed using an NMReady-60e benchtop relaxometer (Nanalysis Corp.) operating at 60 MHz and 1.40 T, whereas the T_2_-weighted MR phantom images were acquired using an Agilent 4.7 T/200 MHz MRI scanning system. The relaxivity measurements and MR phantom tests were conducted threefold and fourfold, respectively.

### MTS Cell Viability Assay

The cytotoxicity effects of PEG-coated magnetite NPs were performed on HeLa, A549, MDA-MB-231 and Jurkat cells using the CellTiter 96**®** AQ_ueous_ One Solution Cell Proliferation Assay (Promega), following our previously reported protocol [41]. Briefly, the cells were cultured in Eagle’s minimum essential medium supplemented with 5% of human platelet lysate (EMD Millipore), 100 μg/mL, 100 U/mL penicillin, streptomycin, and 250 ng/mL amphotericin B (Cellgro) at 37 °C with 5% CO_2_. Cells were plated at a density of ~2 × 10^4^ in 96-well plates and grown until reaching 80–90% confluence [40]. Cell culture medium was then removed and 100 μL of complete medium supplemented with PEG-SPION at different concentrations (ranging from 0.1 to 10 [Fe] mM) was added, and three wells with only fresh complete medium were used as a positive control. The medium was removed after 24 h of incubation and a solution of fresh media containing 20% of CellTiter 96**®** AQ_ueous_ One Solution reagent was added. Wells with fresh complete medium and 3-(4,5-dimethylthiazol-2-yl)-5-(3-carboxymethoxyphenyl)-2-(4-sulfophenyl)-2H-tetrazolium (MTS) reagent without cells were used as a negative control [40]. Cells were then incubated at 37 °C for 30 min. Afterwards, the 96-well plates were centrifuged at 2000 rpm for 10 min. The supernatant was transferred onto a clean microplate and the absorbance at 490 nm was recorded using a Synergy H1 Hybrid Multi-Mode Microplate Reader. The cell viability percentage was determined by means of the following equation: % = [(A_490_ of PEG-SPION-treated cells)/(A_490_ of untreated cells)] × 100. The same protocol was followed when the PEG-SPION/cells and controls were exposed to an external magnetic field (0.2 T). All of the experiments were done in triplicate.

## Results and Discussion

We synthesized PEG-SPIONs free of anchoring groups via a modified co-precipitation approach. The XRD patterns of the as-synthesized products are depicted in Figure S1 (see Additional file 1). The bare XRD patterns of SPION and pure PEG were included for comparison. The diffraction peaks observed in PEG-SPION were indexed to the reflection planes of cubic inverse spinel Fe3O4 corresponding to the (111), (220), (311), (400), (422), (511), and (440) planes, compatible with the JCPDS file no. 79-0418 [41–43]. This indicates that the crystalline structure and phase of SPION are retained by PEG-SPION. Two additional diffraction peaks at 2*θ* = 19.2 and 23.3 that correspond to the crystalline planes of pure PEG were also observed [44], which confirm the successful surface functionalization of SPIONs. The diffraction peaks of PEG-SPIONs are less intense when compared to those of SPIONs, which further supports the adsorption of PEG by SPIONs (wt.% 1:10). The observed well-defined peaks, the absence of secondary phases (including maghemite and other forms of iron oxide) in the XRD patterns, and the co-existence of both phases in PEG-SPIONs are clear indicators of the successful formation of PEGylated Fe_3_O_4_ with high crystalline quality and purity. The broadening of the diffraction peaks of SPION and PEG-SPION is ascribed to their nanocrystalline nature. The calculated average crystal size of SPION was determined by means of Scherrer’s formula, yielding ~16 nm.

**Fig. 1:**
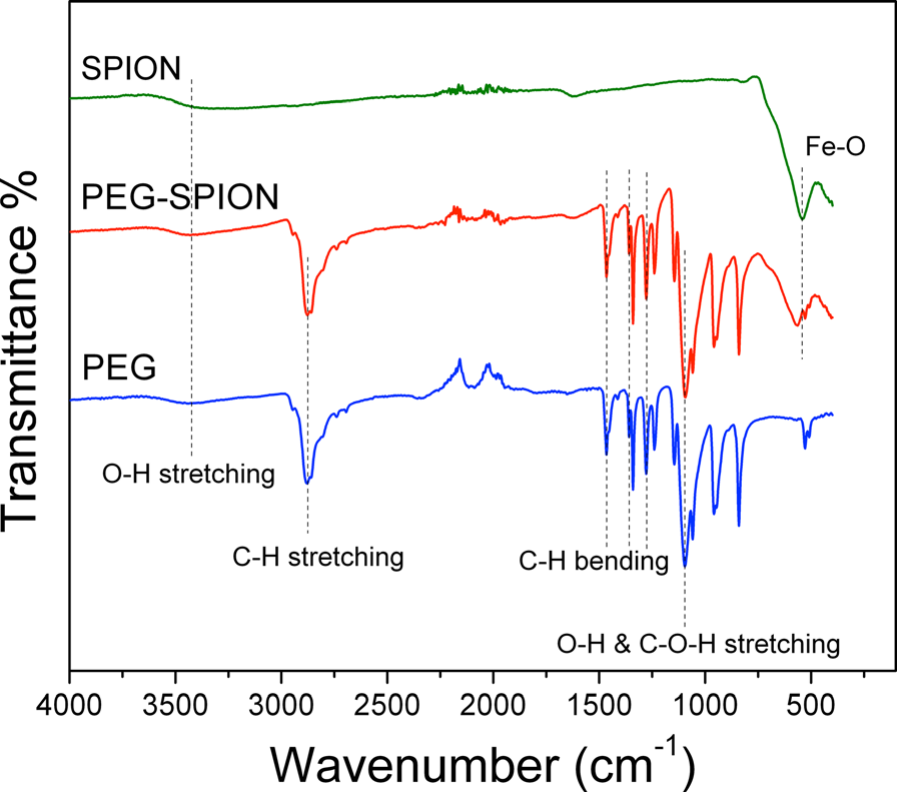
ATR spectra of bare Fe3O4 nanoparticles (SPION), PEGylated Fe3O4 nanoparticles (PEG-SPION), and bare PEG powder

To furnish more evidence of the presence of the PEG layer on SPIONs surface, we conducted ATR analysis. The ATR spectra of SPION, PEG-SPION and PEG are depicted in Fig. 1. The ATR spectrum of PEG shows one main -C-O-C- ether stretch band centered at 1099.3 cm^−1^, and a vibrational band peaked at 1340.4 cm^−1^ that corresponds to the antisymmetric stretch [39]. PEG also exhibits two absorption bands at 1278.7 and 1465.8 cm^−1^ ascribed to the vibration of –CH_2_, and one band at 958.5 cm^−1^ associated to the –CH out-of-plane bending vibration [45]. The ATR spectrum of bare SPIONs shows a prominent vibrational band centered at 541.9 cm^−1^, which is attributed to the Fe-O stretching mode in Fe_3_O_4_. One broad band peaked at ~3437 cm^−1^ for both PEG and SPIONs was also observed and is related to the attached hydroxyl groups [39]. As for PEG-SPIONs, we observed the absorbance of the main ether stretch band centered at 1097.4 cm^−1^, and the –CH_2_ vibrational band peaked at 1276.8 and 1463.9 cm^−1^, indicating the presence of PEG on the SPION surface. The slight redshift observed in these bands is likely due to the effective bonding of the PEG adlayer as a result of the change of chemical environment [46]. In parallel, we have found a shift (21 cm^−1^) of the Fe-O vibration band and was ascribed to the formation of a new band between the PEG coating and SPION surface [39]. We can infer from this analysis that the ether group of PEG is efficiently linked to the positively charged surface of SPIONs, and mediated through a dipole-cation covalent interaction. The transmittance peaks in the range of 1900 to 2300 cm^−1^ correspond to the diamond crystal utilized in the ATR setup.

**Fig. 2:**
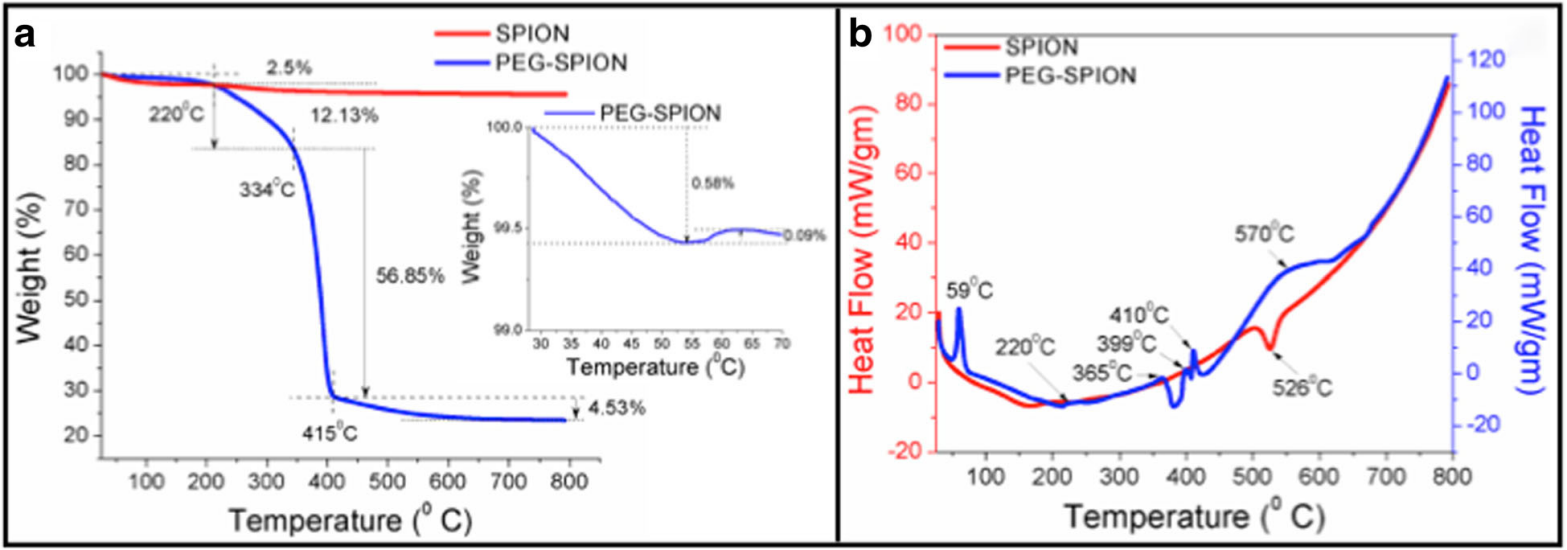
**a** Thermogravimetric analysis curves and **b** differential scanning calorimetry curves of SPION and PEG-SPION. The *inset* shows the weight gain of PEG-SPION

Thermogravimetric analysis (TGA) and differential scanning calorimetry (DSC) were performed to study the thermal behavior of SPION and PEG-SPION, which further confirms the suitable PEGylation. The corresponding curves are illustrated in Fig. 2. As seen in the first stage in Fig. 2a, a subtle weight loss of 2.5% of SPION and PEG-SPION in the region of 30–220 °C is ascribed to the loss of adsorbed water molecules from their surface. The weight loss was observed to be more rapid in SPION than in that of PEG-SPION. In addition, Fig. 2a (inset) shows a marginal weight gain of PEG-SPION at 59 °C, and was confirmed by an exothermic peak as shown in the DSC analysis curve in Fig. 2b. The actual reason for this increase in weight is unknown since the conversion of Fe_3_O_4_ to γ-Fe_2_O_3_ is very unlikely at this temperature. Above 220 °C, only 1.5% of weight loss was observed for SPION up to 800 °C yielding an overall weight loss of 4%. But in case of PEG-SPION, a gradual weight loss of 12% in the temperature range of 220–334 °C followed by a rapid weight loss of 57% in the temperature range of 334-415 °C, and again a gradual weight loss of 5% from 415 to 600 °C was seen. These stepwise weight losses are remarkably observed in the DSC curve (Fig. 2b) with multiple exothermic peaks between 220 and 600 °C. This significant weight loss of 73% indicates the decomposition of PEG moieties. To calculate the grafting density of PEG on SPION, we assume that SPION is spherical in shape with a radius of 5.5 nm with a surrounding PEG layer (MW_PEG_ = 3350 gm/mol). Using the following equation [47]:

3 $$ {\sigma}_{TGA} = \frac{\frac{wt{\%}_{PEG}}{wt{\%}_{SPION}}\ {\rho}_{SPION\ \left(\frac{4}{3}\pi {r}_{SPION}^3\right){N}_A}}{M{ W}_{PEG}\  4\pi {r}_{SPION}^2} $$

where *ρ*_SPION_ and N_A_ are the density of SPION (5.24 g/cm^3^) and Avogadro’s number, respectively, and introducing the respective values we obtained a grafting density of ~5 chains/nm^2^. This relatively high value implies that the PEG chain is densely packed on SPION core, as expected, and responsible for the steric repulsion in the solution.

**Fig. 3:**
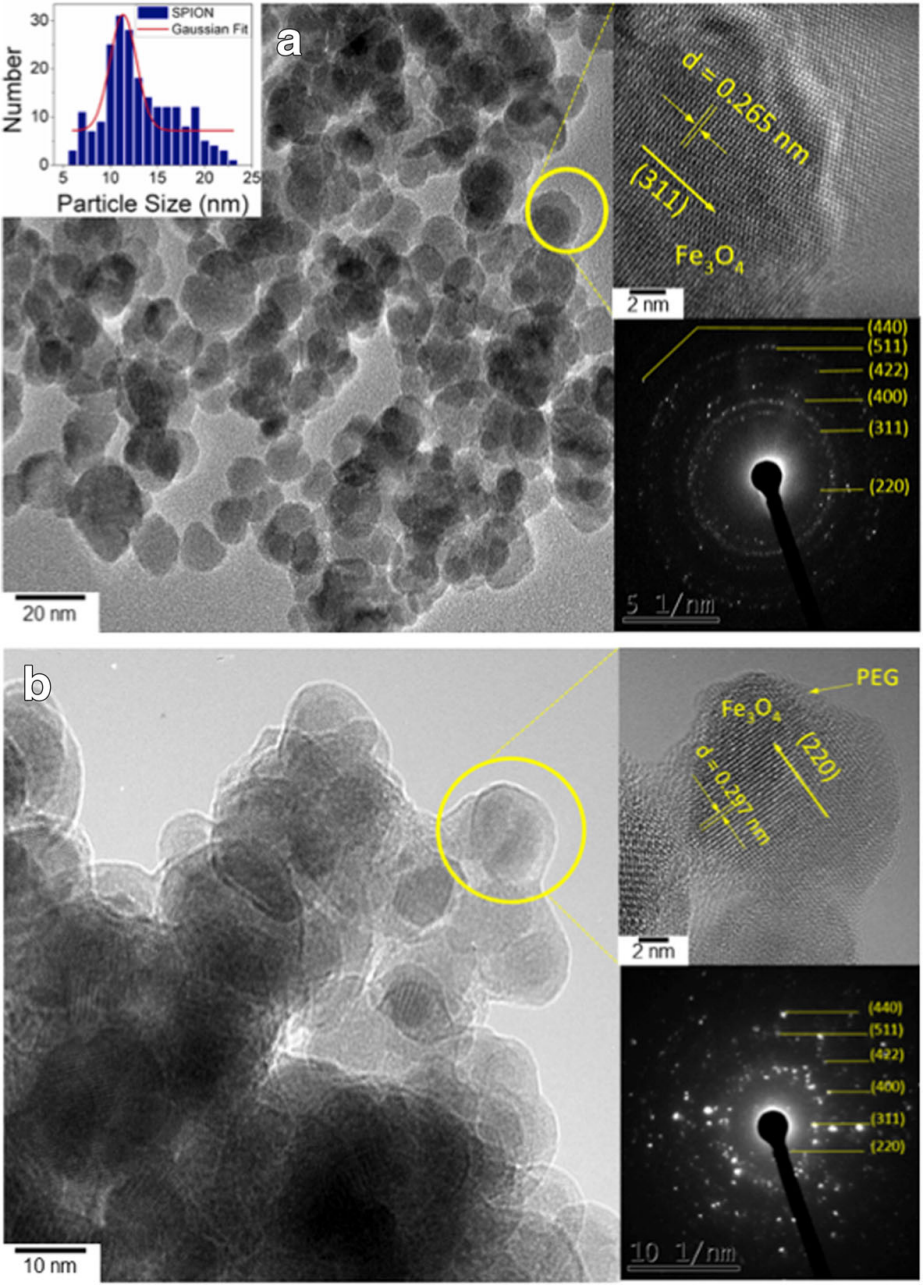
**a** Bright field HRTEM images of bare Fe3O4 nanoparticles (SPION). The *top left* and *right* and *bottom left* insets show the average crystal size distribution, close-up and the SAED pattern of SPION, respectively. **b** Bright field HRTEM images of PEGylated Fe3O4 nanoparticles (PEG-SPION). The *top left* and *right* and *bottom left insets* show the average crystal size distribution, close-up, and the SAED pattern of PEG-SPION, respectively

The morphology, size, and crystallinity of the products were studied by electron microscopy. HRTEM images of SPION and their corresponding crystal size distribution and selected area electron diffraction (SAED) are depicted in Fig. 3a. From the gray scale contrasts, the nanostructures appear to be homogeneously distributed on the Cu-grids, and composed of many near-spherical NPs. The agglomeration observed in this thin cluster could be attributed to the mutual magnetic attraction among the NPs. A closer look (lattice-resolved image in top right inset) reveals that the surface of individual NPs is clean and smooth, without any sheathed amorphous phase. An interplanar spacing of 0.26 nm corresponding to the (311) plane of Fe_3_O_4_ was also identified. The crystal size distribution obtained by statistical image analysis (see top left inset) shows that the crystal diameters are in the 6–23 nm range, with an average size of ~11 nm, compatible with that estimated by the XRD analysis. The SAED pattern (taken on the region shown in Fig. 3a) depicted in the bottom left inset was indexed to polycrystalline -cubic Fe3O4 [(400), (511), (422), (400), (311), and (220)], consistent with the bulk XRD results.

Figure 3b shows the HRTEM images and SAED patterns of PEG-SPION. Our observations indicate that when the capping ligand (PEG polymer) is added, the SPION cores retain their morphology, phase, narrow size distribution and crystallinity. Note that the irradiation of the 200-kV electron beam did not cause any damage, phase transformation or amorphous carbon deposition on the PEG-SPIONs. The agglomerated feature observed in PEG-SPION is associated to the drying process of the dispersions. Their relatively uniform dispersion in water is correlated to the effective PEG capping process (as displayed in Figure S2, see Additional file 1). This fact was confirmed by the HRTEM image displayed in the top right inset, in which a less-crystalline phase that surrounds the cores’ surface is easily differentiated by contrast. Thus, one would expect a bigger particle size at this PEG (3350)/SPION ratio (wt.% 1:10, PEG with mol. wt. > 3000 given the dense coating over the NPs surface) [31], which indicates the formation of adlayers on single particles. This was confirmed by the DLS measurements, which will be discussed in the next section.

The linkage of PEG onto the surface of SPIONs was further corroborated by DLS and zeta potential measurements. The DLS profiles of bare SPION and PEG-SPION in water (at different pH values) and different solvents are depicted in Figure S3 (see Additional file 1). From this Figure, the hydrodynamic diameter (D_H_) of SPIONs was observed to be higher than that of PEG-SPION in most of the solvents. At neutral pH, the observed D_H_ is 145.8 nm (PDI = 0.28) and 115 nm (PDI = 0.154) in water for SPION and PEG-SPION, respectively. These sizes are much higher than those observed in HRTEM. This is ascribed to the agglomeration of SPION as a result of their mutual magnetic attraction, whereas the formation of brush-type configuration of PEG (due to the high grafting density of 5 chains of PEG per nm^2^ surface area of SPION and large Flory radius of 4.7 nm) may partly contribute to the increase in hydrodynamic diameter for PEG-SPION. In acidic and basic conditions, the induction of surface hydration or the formation of electric double layer (EDL) on the surface of dispersed PEG-SPION may lead to the increased hydrodynamic diameter [48, 49]. Further, when dispersed in biological buffers, such as PBS 7.2 and NaCl solution, both SPION and PEG-SPION tend to flocculate due to Debye screening of electrostatic repulsion or adsorption of counter-ions [50].

The zeta potential measurements as a function of pH for SPION and PEG-SPION are shown in Figure S4 (see Additional file 1). It was observed that SPION acquire positive charges even at neutral pH with zeta potential of +2.15 mV, which gradually increases to +24.2 mV at acidic conditions (pH = 3). This clearly suggests the presence of positive charges on the surface of SPION and the preferential dissolution or deposition of H^+^ co-ions in acidic medium [51]. The curve with solid circles in Figure S4 shows that the zeta potential of SPION almost drops to 0 mV at pH > 7 and pH < 8, and the SPION aggregate drastically. But an opposite trend was observed in basic conditions acquiring zeta potential of −37.7 mV at pH = 11. This particle stability is provided by electrostatic repulsion. Further, in case of PEG-SPION, the –OH group of the PEG is protonated in acidic medium (pH = 3) giving zeta potential of +10.8 mV, as shown in solid square in Figure S4. Below this pH, it was observed that the –OH group of PEG was gradually deprotonated and the isoelectric point (IEP) was determined to be at pH ~ 4.1 where the zeta potential becomes 0 mV. Similarly, the deprotonation of –OH group causes the formation of negative charges around the surface of PEG-SPION giving rise to large negative zeta potential at neutral and basic pH. The highest stability of PEG-SPION was noted at pH 11 with zeta potential of −30.6 mV, which is attributed to the electrostatic repulsion.

**Fig. 4:**
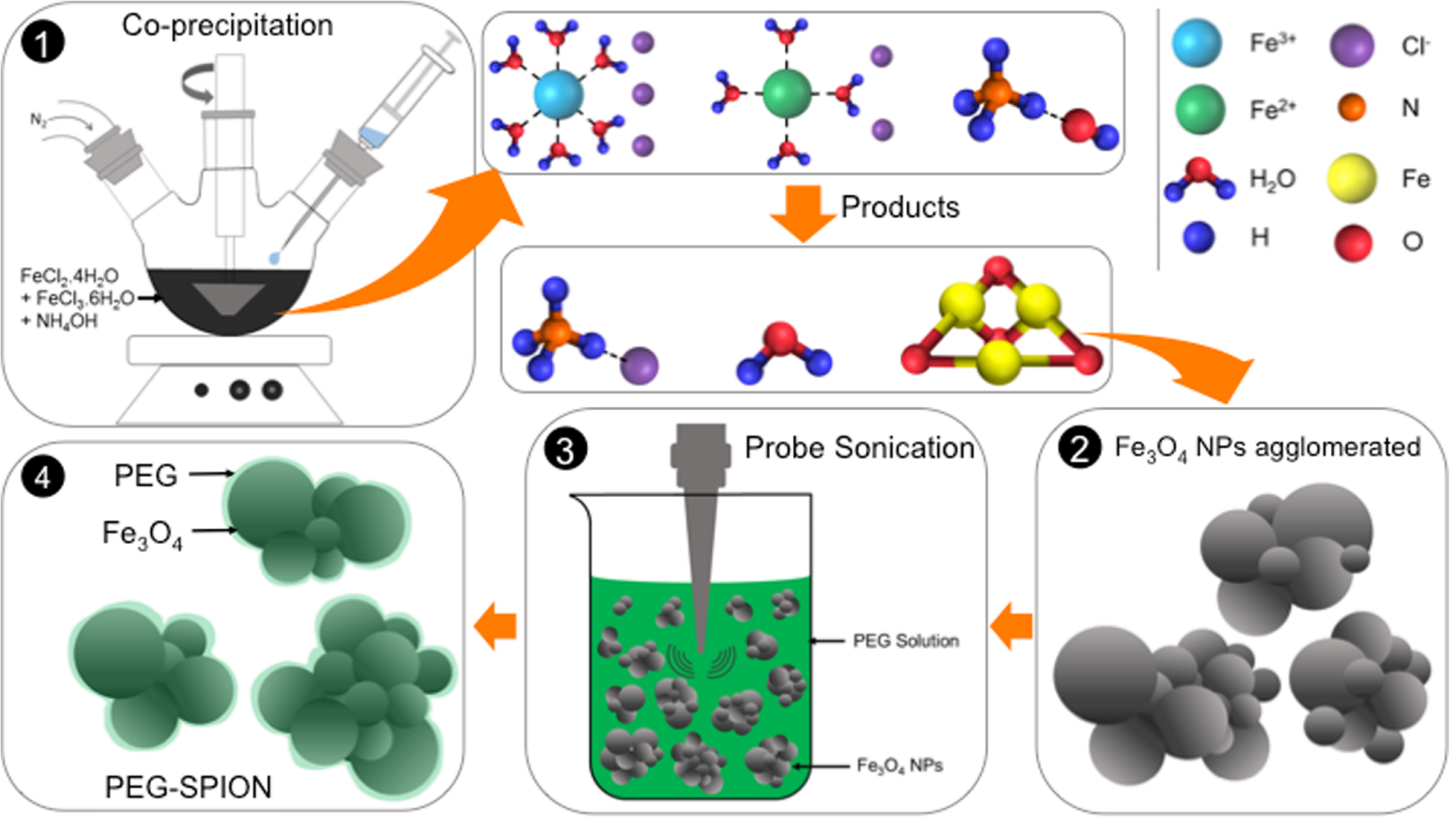
Schematic representation of the growth mechanism of PEG-SPION, which can be visualized as a core-shell system consisting of a superparamagnetic core and a magnetically disordered shell

Taken altogether, a plausible growth mechanism can be explained as follows: (i) The first step involves the co-precipitation method for the synthesis of SPION from reaction of aqueous mixture of iron salts (molar ratio of Fe^3+^/Fe^2+^ set at 2) under strong alkaline conditions. This results in SPION that are not soluble in water; (ii) The Fe_3_O_4_ NPs agglomerate due to high surface energy/unsaturated coordination sites of surface atoms as well as due to the magnetic interaction of the NPs with each other; (iii) Probe sonication allows the dissolution of NPs by breaking intermolecular interactions and homogeneously dispersing them in PEG solutions; (iv) Dipole-cation covalent binding between the ether group of PEG and positively charged surface of magnetite is responsible for the formation of PEG-coated Fe_3_O_4_ nanoparticles, which are highly dispersible in aqueous medium. This growth mechanism is illustrated in Fig. 4. And the proposed mechanism of dipole cationic binding of ether group of PEG to SPION surface and hydration process of PEG for aqueous dispersibility is illustrated in Figure S5 (see Additional file 1).

**Fig. 5:**
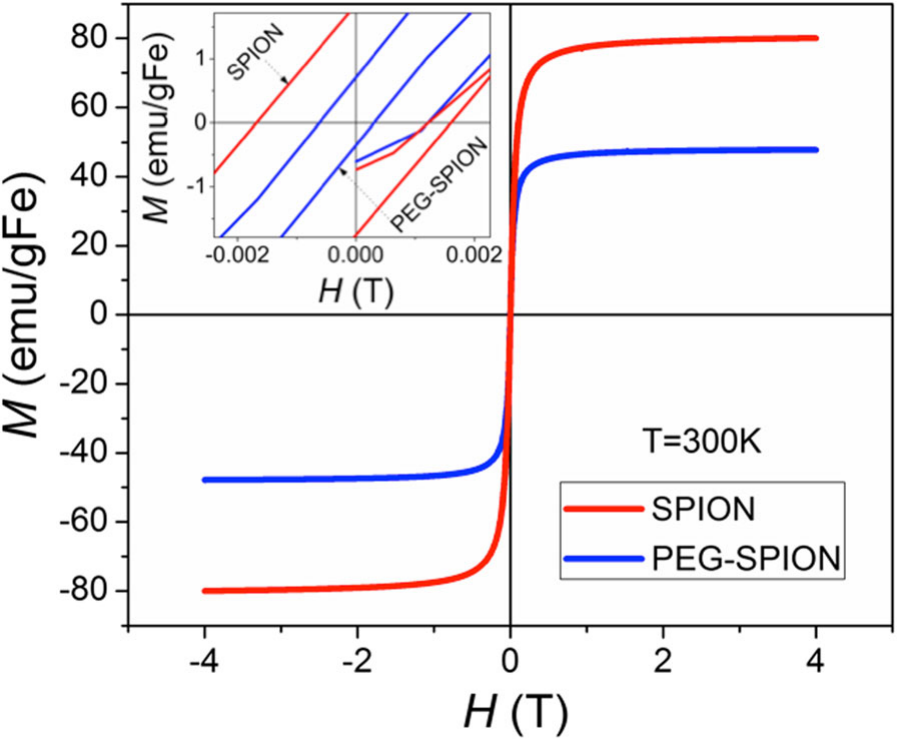
M-H hysteresis curves of SPION and PEG-SPION. The *inset* shows the values of MR and HC for SPION and PEG-SPION at low magnetic fields

To identify whether these water-soluble NPs are superparamagnetic, we conducted dynamic vibrating sample magnetometry and static field/temperature dependent magnetization measurements. The magnetic hysteresis loops of bare SPIONs and PEG-SPIONs collected at room temperature and up to 4 T are depicted in Fig. 5. The well-defined *M*–*H* curves show that the PEG-SPIONs exhibit a marked magnetic response (inherited from the SPION cores) with a saturation magnetization (*M*_S_) of 48 emu/gmFe, which represents 60% of that found for bare SPION. A reduction of the remnant magnetization (*M*_R_) and coercivity (*H*_C_) from 1.61 to 0.54 emu.gm^−1^ and from *μ*_o_ *H*_C_ = 1.66 to 0.61 mT, respectively, was observed after the PEGylation (see inset of Fig. 5). The fact that PEG-SPION are rapidly magnetized when exposed to an external magnetic field, and that they exhibit a small *H*_C_ with a near-zero *M*_R_ (very low volume anisotropy), show that they are superparamagnetic [42], which is consistent with the single-domain superparamagnetic criteria for Fe_3_O_4_ at this size regime (average core size ~11 nm). The reductions in *M*_S_, *M*_R_, and *H*_C_ could be related to the surface effect or to the change in shape and size [52]. Since there are no significant changes in shape and size of SPIONs after PEGylation, the reduction of *M*_S_ is ascribed to the presence of the diamagnetic layer of PEG and to the spin-canting effect, i.e., lack of full alignment of the spins in surface atoms [52]. In other words, the reduction of *M*_*S*_ may be ascribed to weight contribution from the nonmagnetic PEG in the composite as described by Tian et al. [53].

**Fig. 6:**
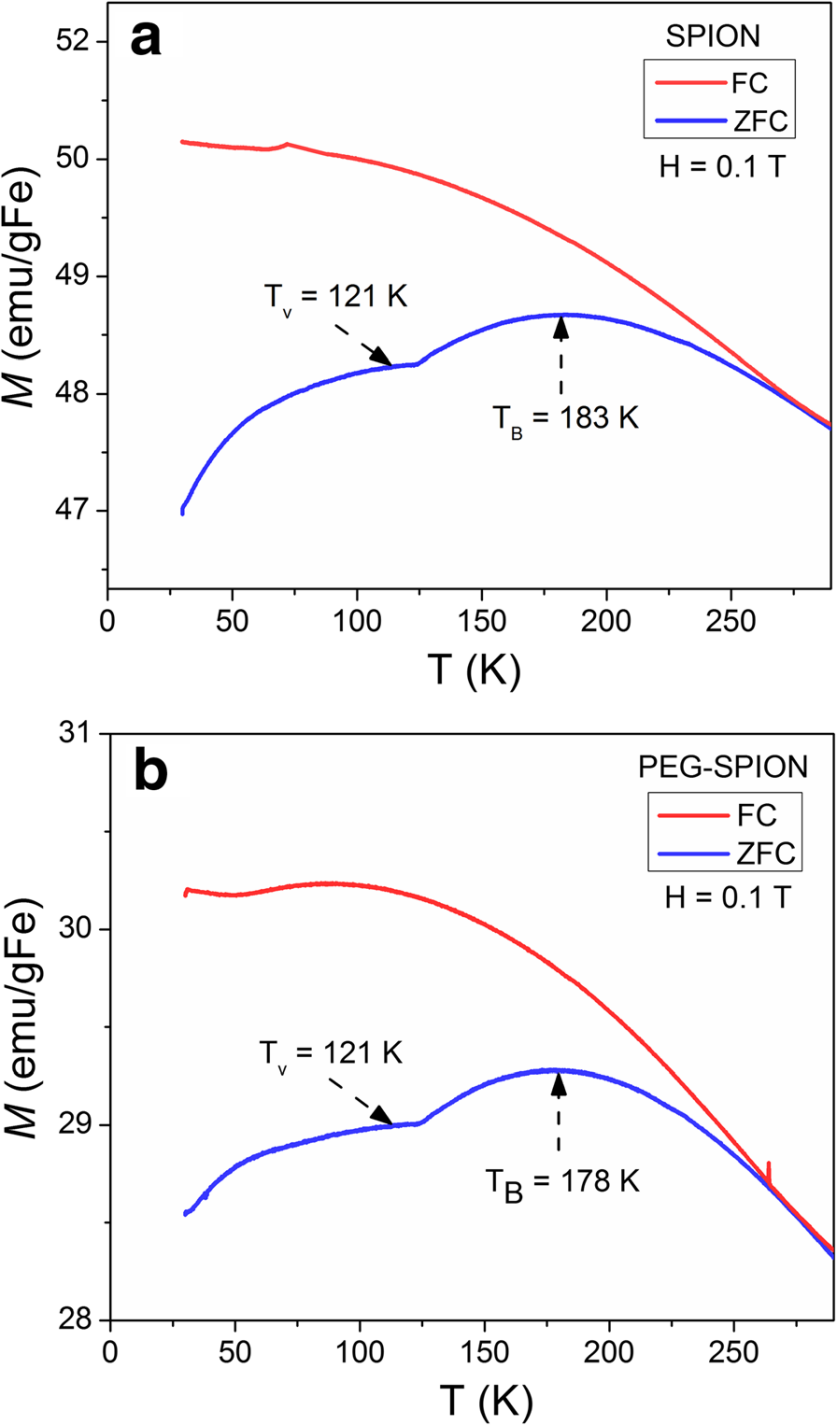
ZFC and FC profiles of **a** SPION and **b** PEG-SPION measured at 30–300 K

The temperature (30–300 K) dependent magnetization curves of SPION and PEG-SPION are depicted in Fig. 6. The zero-field-cooled (ZFC) and field-cooled (FC) magnetization curves yield the blocking temperatures (*T*_B_) of SPION and PEG-SPION 183 and 178 K, respectively. From these curves, prominent and flat plateaus centered at ~ *T*_B_ were observed, which are associated to the superposition of ZFC curves (having different peak points) of different-sized NPs. This is correlated to the crystal size distribution found in the HRTEM analysis. We also observed that the Verwey transition temperature (*T*_V_) is at ~121 K for both SPION and PEG-SPION (this temperature is typical in Fe_3_O_4_). *T*_V_ is the characteristic transition temperature for magnetite, above which its crystallographic phase is transformed from monoclinic to cubic symmetry, and below which it becomes electrically insulating [54]. In fact, Fe_3_O_4_ is a mixed valence compound in which the Fe^3+^ and Fe^2+^ ionic pattern at octahedral sites changes (at ~ *T*_V_) from a long-range low-temperature charge ordering to a high-temperature dynamic disorder [55–57]. Taken altogether, PEG-SPION are water-dispersible and show a well-defined superparamagnetic feature at room temperature (similar to those reported for intermediate biomolecule anchored PEG-SPION), which is crucial for MRI applications.

**Fig. 7:**
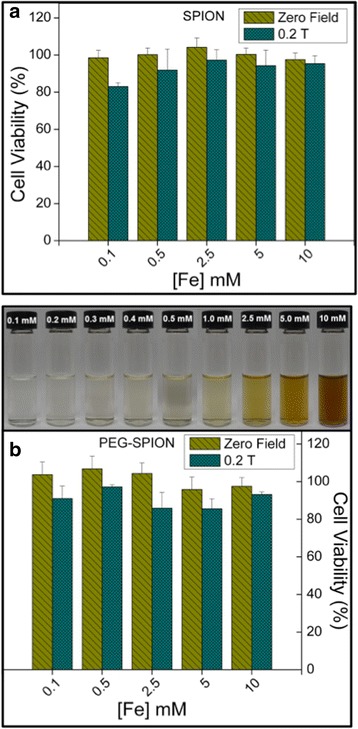
In vitro MTS cytotoxicity assays showing the HeLa cell viability in the presence of SPION (**a**) and PEG-SPION (**b**) at different concentrations after incubation for 24 h, and in absence and presence of an external static magnetic field. The *upper inset* in **b** shows the optical images of PEG-SPION in solution at different concentrations

Prior to evaluating PEG-SPION as a MRI *T*_2_ CA, we studied their cytotoxic effect on cervical adenocarcinomic HeLa cells, A549 lung cancer cells, MDA-MB-231 breast cancer cells, and T lymphocyte Jurkat cells. Cell viabilities were examined through the measurement of cell metabolic activity. The mitochondrial function was measured via MTS viability assay after incubating the cells with different concentrations (0.1–10 [Fe] mM) of bare SPION and PEG-SPION for 24 h. Viable cells convert MTS tetrazolium into formazan dyes, which can be spectrophotometrically detected [43]. Since the MRI CAs-based formulations used in clinical trials are exposed to external magnetic fields, we have also evaluated the cytotoxicity of bare SPION and PEG-SPION on cells when influenced under a static magnetic field (continuous 1-h exposition and strength ~0.2 T). The HeLa cell viability results are depicted in Fig. 7. In this range of concentrations (0.1–10 [Fe] mM), SPION and PEG-SPION showed no toxic response on human cancer cells in the absence of the field. Similar results were recently reported by Kim et al. working with 12-nm sized iron oxides capped with PEG-derivatized phosphine oxide ligands, and MCF-7 human cells [52]. However, when the static field is turned on, the average cell viability slightly decreased until a value of ~92% for bare SPION and ~91% for PEG-SPION.

**Fig. 8:**
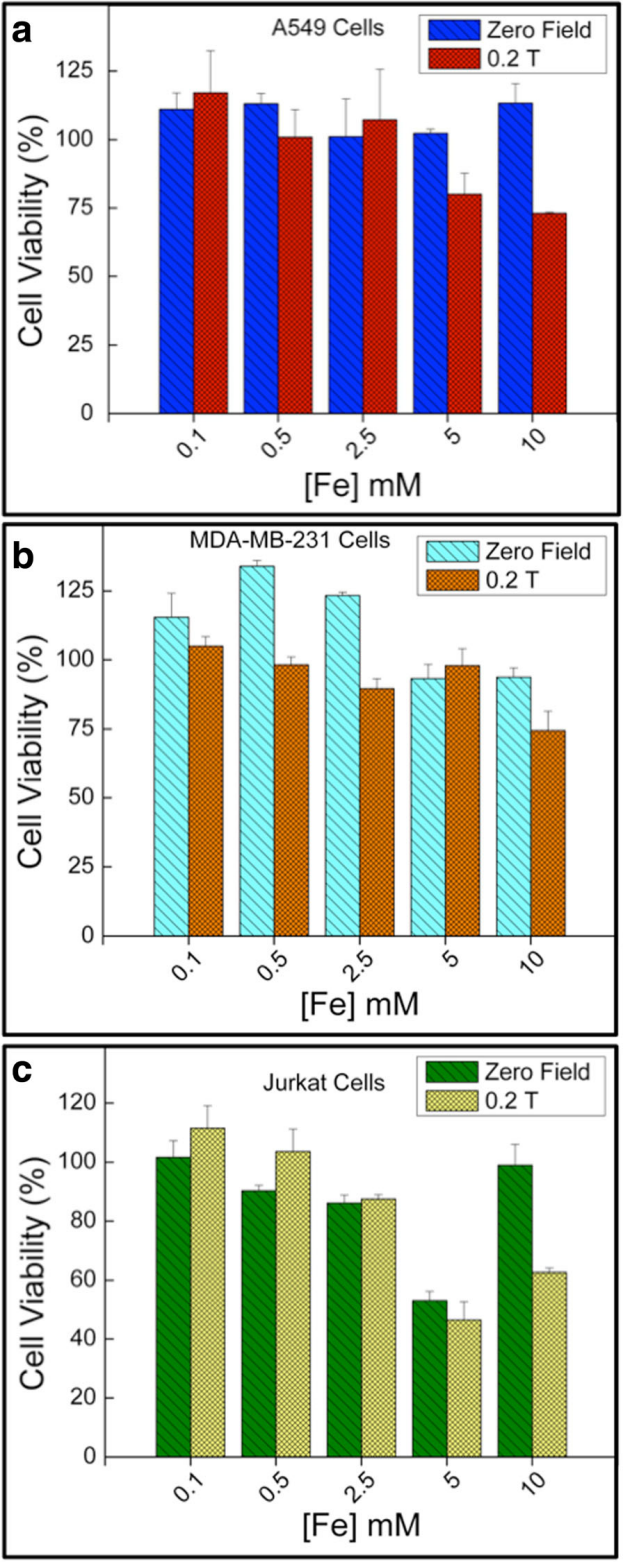
In vitro MTS cytotoxicity assays showing the A495 (**a**), MDA-MB-231 (**b**) and Jurkat (**c**) cell viabilities in the presence of PEG-SPION at different concentrations after incubation for 24 h, and in absence and presence of an external static magnetic field

These findings reflect that PEG-SPION causes negligible cytotoxicity (~9% of reduction in cell viability) even at iron doses as elevated as 10 mM, which is higher than that typically used in nanoparticle-based MRI [58]. It is noteworthy that under the influence of a static 0.2 T field, the cell viabilities of different line cells (such as human neuronal FNC-B4 and breast carcinoma cells) are unaffected, even at more intense fields (1.5 T) the growth of HeLa cells is unaltered [59]. Nonetheless, when the effect of the field (~0.1 T) is combined with that of SPION, more adverse changes in the integrity of cancer U937 cells can be induced, when compared to normal peripheral blood mononuclear cells, which are associated to the diamagnetic nature of the biological cells [60]. The results indicate that although the SPION are capped with a diamagnetic PEG layer, no toxic effect is detected, compatible with the report of the World Health Organization that points out that there is no health effect at low static magnetic fields [61]. We also observed that PEG-SPION are tolerable (unchanged cell viability) to A549, MDA-MB-231 and Jurkat cells in the range of 0.1–2.5 [Fe] mM, when subjected to the field under similar exposition conditions (see Fig. 8). However, when the iron concentration was further increased (>2.5 mM and up to 10 mM), the average cell viability decreased in 20–50% of its initial value, which could have been induced by slow apoptotic cell death because of the cell membrane damage and acute cell injury that occurs when the nanoparticle clusters are in close proximity to the cell membrane. It is assumed that the cellular activity is further activated when the field is turned on (during incubation of PEG-SPION with A549, MDA-MB-231 and Jurkat cells), enabling the SPION to affect the cell metabolism. Our findings are compatible with recent reports by Z. Coker [62] et al. and K. Urbas [63] et al, in which at high Fe_3_O_4_ doses (~100–600 μg/mL) a drastic reduction of cell viability (~90–10%) of L929 fibroblasts and CHO-K1 cells is observed, thus establishing a correlation between the field (as low as ~2–10 mT) and the mitochondrial activity [62, 63]. Nevertheless, this huge range of concentrations is not practical, signifying that for in vitro and in vivo MRI applications the PEG-SPION are not cytotoxic to four different line cells. The variance (ANOVA) test indicated that there are no statistically significant differences attributed to PEG-SPION concentrations (*p* = 0.1457) for all the measurements presented in this work.

**Fig. 9:**
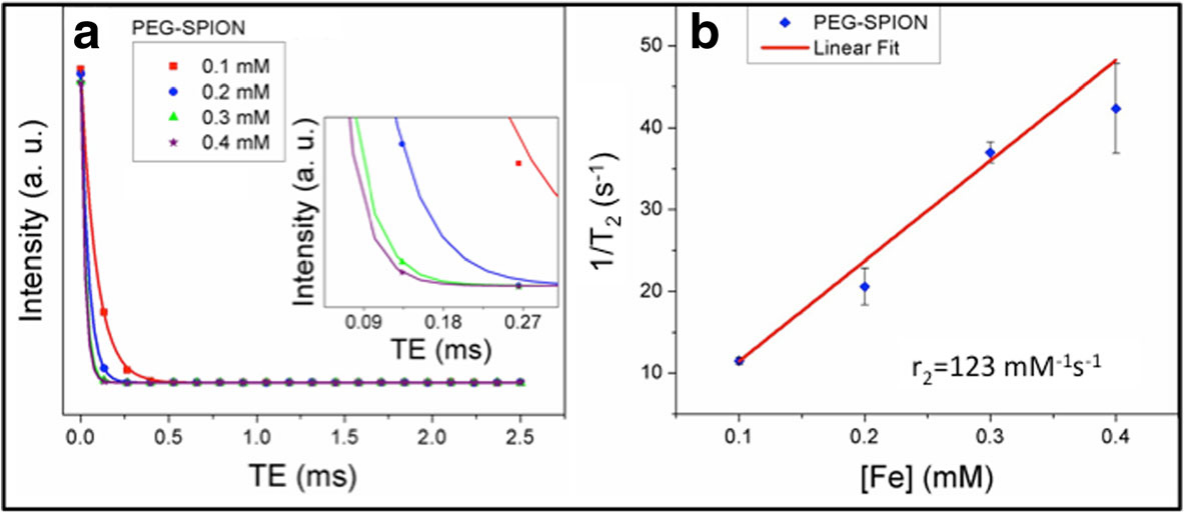
**a** Transverse intensity vs echo time curves and **b** T2 relaxation rate as a function of iron concentrations for PEG-SPION at human body temperature

To evaluate the feasibility of using PEG-SPION as MRI *T*_2_ CA, we measured the proton relaxation time of PEG-SPIONs for different iron concentrations (0.1–0.4 mM) at human body temperature. The transverse intensity vs echo time curves are depicted in Fig. 9a. The relaxivity (*r*_2_ expressed in mM^−1^s^−1^) is calculated as the slope of the linear plot of the relaxation rate (*R*_2_ = 1/*T*_2_) vs [Fe] mM, and is displayed in Fig. 9b. It can be observed that the transverse intensity sharply declines with the echo time, indicating that the enhanced transverse relaxation of protons increases with the iron concentration (see inset of Fig. 9a), as expected. The linear dependency of transverse relaxation yields *r*_2_ = 123 ± 6 mM^−1^s^−1^, which is moderately higher than iron oxide nanospheres, nanoworms and Feridex (with range of sizes similar to those reported here) [10], and compatible with that reported for 14-nm SPIONs capped with 2000–5000 Da PEG [39] (in the present study, PEG = 3350 Da). It is well-known that the susceptibility-induced *T*_2_-reduction is due to the dephasing of magnetic moments of the protons in water molecules, which is caused by the field gradients created by small magnetized NPs. Magnetic NPs induce spin-spin relaxation as protons pass through such field gradients. For a spherical bare nanoparticle with a specific core size, the higher field gradient takes place close to the vicinity of its core. Thus, when the protons of water molecules approach close to the nanoparticle, the coating layer hampers to some extent that the water molecules approach to higher field gradients (close to the core), i.e., it behaves as an impermeable coating. Nevertheless, we observed that in PEG-SPION (with a *M*_S_ of ~40 emu.gm^−1^) the PEG coating is neither compact nor solid, and is thin enough such that the water molecules attain higher translational diffusion, thereby being exposed to a wide zone of high field gradients, which is effectively time-averaged [64]. Thus, the proton relaxation in PEG-SPIONs is slower than that of bare SPIONs, consistent with our findings.

**Fig. 10:**
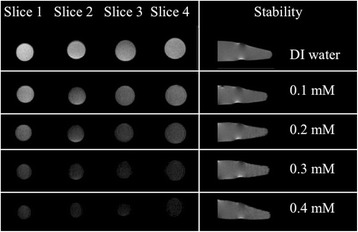
T2-weighted MR phantom images of PEG-SPION in aqueous solution at human body temperature and using a 3.7-T MRI scanner, showing a low degree of sedimentation (range of 0.1–0.4 [Fe] mM) at the *bottom* of the aliquots

**Fig. 11:**
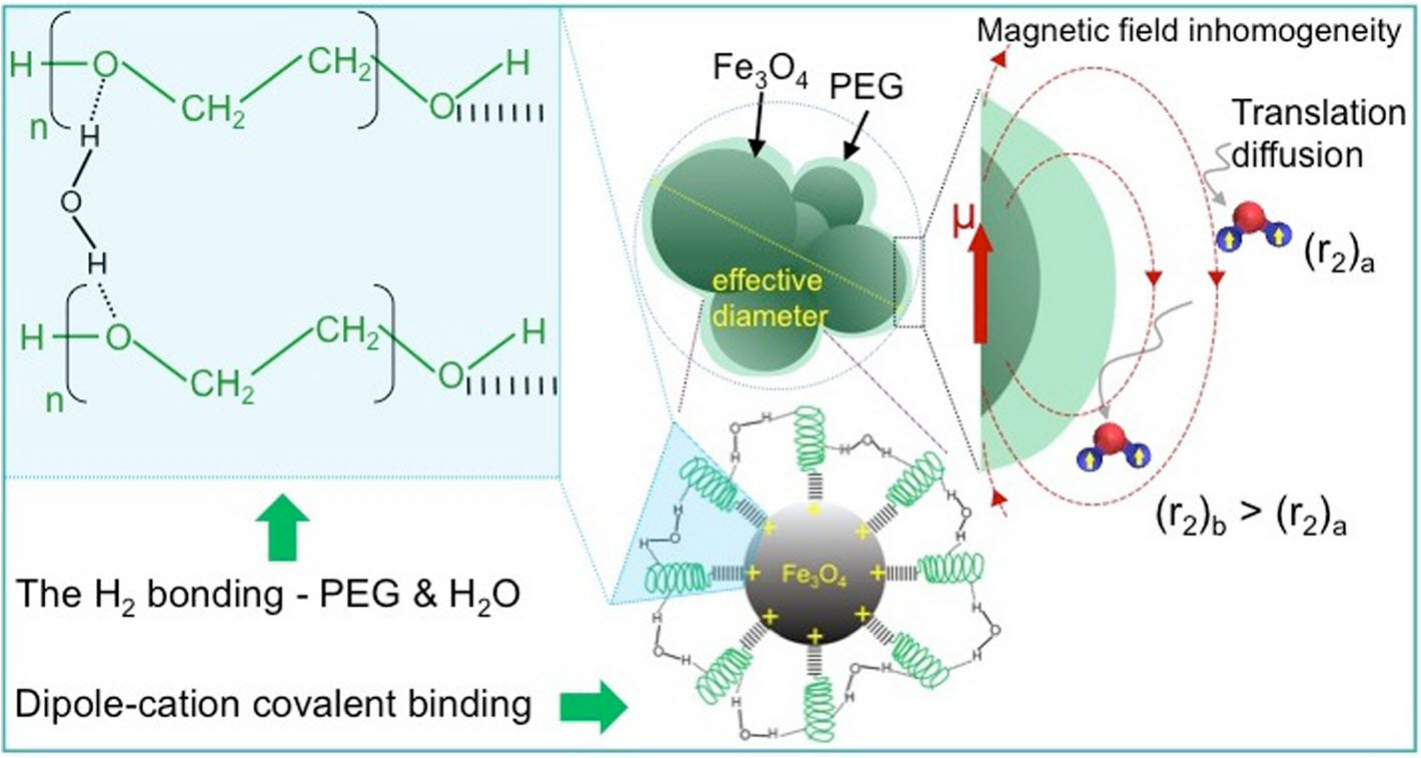
Schematic illustration of the enhanced contrast mechanism of PEG-SPION

Figure 10 shows the *T*_2_ -weighted MR phantom and stability tests of PEG-SPIONs in aqueous solution at different iron concentrations (0.1–0.4 mM) taken at human body temperature. The *T*_2_-weighted MR phantom test of deionized water was included for comparison. The images obtained from four different slices of single aliquots (labeled as Slice 1, Slice 2, Slice 3, and Slice 4), show contrast enhancement as the iron concentration increases. This enhancement is ascribed to their higher magnetic moment that induces strong magnetic inhomogeneity around the NPs causing the distortion of spin coherence of water protons in transverse relaxation. In addition, it is well-established that *T*_2_ is highly dependent on both the *M*_S_ and the effective radius of the core (based on the quantum mechanical outer sphere theory) [65], and given that *M*_S_ is reduced to 54% of its initial value, one can thus assume that the most critical point to enhance the contrast is to increase the effective boundary radius of PEG-SPION (as shown in eq. 2). Our electron microscopic and spectroscopic analyses show a structural increase of ~15 times because of the nanoparticle clustering. Similar results were recently reported by Zhao and co-workers using concave octapod iron oxides NPs, which showed higher relaxivity values (when compared to those of spherical SPION) attributed to the increase of the effective radius that results from their unique morphology [65]. We expect that the PEG-assisted nanoparticle clustering induces a more inhomogeneous (compared to bare SPION) local magnetic field under the influence of the external field, which can further induce proton dephasing and enhance the *T*_2_ shortening. A depiction of this plausible relaxation mechanism is illustrated in Fig. 11. Finally, our findings suggest that PEG molecules may immobilize water molecules over large regions [36], with a low degree of sedimentation (as shown in Fig. 10), and the size of PEG-SPION would be beneficial for increasing blood half-life (~few tens of minutes in SPION) for in vivo MRI tests by avoiding uptake by macrophage cells [59], signifying that the NPs can escape from the immune system. However, further studies to evaluate the potential of this robust PEG-SPION system as an efficient *T*_2_ CA in vivo MR imaging are needed. The proposed enhanced contrast mechanism is further detailed in Fig. 11, and the comparison of the *r*_2_ relaxivity with previous studies is also presented in Table 1.

**Table 1:** Comparison of *r*_2_ relaxivity with the current study

Core material/size	Shell material	*Z* ave. size (nm)	*r*_2_ (mM^−1^s^−1^)	*B*_0_ (*T*)	References
Fe_3_O_4_/6 nm	PEG-phosphate	−	42	1.41	[66]
γ-Fe_2_O_3_/−	APTMS	16	54.7	−	[67]
γ-Fe_2_O_3_/−	Porous SiO_2_	27	64	−	[68]
Fe_3_O_4_/5.85 nm	Dextran	30	65	1.5	[69]
γ-Fe_2_O_3_/−	Citrate/Liposomes	300	67	−	[70]
Fe_3_O_4_/8.5 nm	PEG	93	76.2	1.5	[71]
Fe_3_O_4_ /11 nm	PEG	115	123	1.41	This study

## Conclusions

We synthesized 11-nm SPION PEGylated without intermediate biomolecules that show enhanced *T*_2_ relaxivity. The key strategy for this successful PEGylation is the existence of a dipole-cation binding (either covalent or ionic) between the ether group of PEG and the positively charged surface of the Fe_3_O_4_ cores. These hydrophilic NPs retain the morphology of the cores, and exhibit a good colloidal stability in both aqueous media and physiological environments due to the hydrogen bonding of water molecules to electron-rich oxygen atoms in the PEG chain. Our results indicate that the PEG-SPION generate MRI contrast enhancement (per-Fe atom relaxivity ~123 mM^−1^s^−1^) on *T*_2_-weighted sequences, and hence can be considered as highly sensitive *T*_2_ CA. This enhancement is correlated to the increased effective radii due to nanoparticle clustering and the local field inhomogeneity of the magnetic core. The fact that the anchor-free PEG-SPION do not require increased saturation magnetization values to produce high magnetic relaxivities can be a key in the development of next-generation CAs for nascent-stage cancer diagnosis. These findings have broader significance, since they can be extended for the effective conjugation of the NPs with a variety of macromolecules, making them suitable candidates for enhancing the signal-to-noise ratio in MRI and obtaining refined anatomical images.

## Additional file

Additional file 1: Figure S1. XRD patterns of bare Fe_3_O_4_ nanoparticles (SPION), PEGylated Fe_3_O_4_ nanoparticles (PEG-SPION) and pure PEG powder. **Figure S2.** (a-e) Bright field HRTEM images of PEG-SPION showing Fe_3_O_4_ cores fully coated with PEG. **Figure S3.** Zeta potential measurements showing the isoelectric point (IEP) of SPION and SPION stabilized with PEG (3350). **Figure S4.** DLS measurements of SPION and PEG-SPION dispersed in deionized water. **Figure S5.** A proposed mechanism of dipole cationic binding of ether group of PEG to Fe_3_O_4_ surface and hydration process of PEG for aqueous dispersibility. (DOCX 71194 kb)

## Abbreviations

APTMS: Aminopropyltrimethoxysilane
ATR: Attenuated total reflectance
CA: Contrast agent
DLS: Dynamic light scattering
HRTEM: High-resolution transmission electron microscope
IEP: Isoelectric point
MRI: Magnetic resonance imaging
SAED: Selected area electron diffraction
SPION: Superparamagnetic iron oxide nanoparticles
XRD: X-Ray diffractometer
