# Supplementary material for: Enhanced MRI T2 Relaxivity in Contrast-Probed Anchor-Free PEGylated Iron Oxide Nanoparticles
Source: Nanoscale Res Lett. 2017 Apr 27;12:312. doi: 10.1186/s11671-017-2084-y (PMC5407416; doi:10.1186/s11671-017-2084-y)
Supplement: Additional file 1: Figure S1. — XRD patterns of bare Fe3O4 nanoparticles (SPION), PEGylated Fe3O4 nanoparticles (PEG-SPION) and pure PEG powder. Figure S2. (a-e) Bright field HRTEM images of PEG-SPION showing Fe3O4 cores fully coated with PEG. Figure S3. Zeta potential measurements showing the isoelectric point (IEP) of SPION and SPION stabilized with PEG (3350). Figure S4. DLS measurements of SPION and PEG-SPION dispersed in deionized water. Figure S5. A proposed mechanism of dipole cationic binding of ether group of PEG to Fe3O4 surface and hydration process of PEG for aqueous dispersibility. (DOCX 71194 kb) [file 11671_2017_2084_MOESM1_ESM.docx]

**Enhanced MRI *T*_2_ Relaxivity in Contrast-Probed Anchor-free PEGylated Iron Oxide Nanoparticles**

Bibek Thapa*^,^**^†, ╫,^** ^¶^**,** Daysi Diaz-Diestra**^§, ╫,^** ^¶^**,** Juan Beltran-Huarac***^,†, ╫^**, Brad R. Weiner**^§, ╫,^**^¶^ and Gerardo Morell**^†, ╫,^** ^¶^

**^†^**Department of Physics, University of Puerto Rico, San Juan, PR 00931, USA

**^╫^**Molecular Sciences Research Center, University of Puerto Rico, San Juan, PR 00926, USA

^¶^Institute for Functional Nanomaterials, University of Puerto Rico, San Juan, PR 00936, USA

**^§^**Department of Chemistry, University of Puerto Rico, San Juan, PR 00931, USA

**Figure S1.** XRD patterns of bare Fe_3_O_4_ nanoparticles (SPION), PEGylated Fe_3_O_4_ nanoparticles (PEG-SPION) and pure PEG powder.


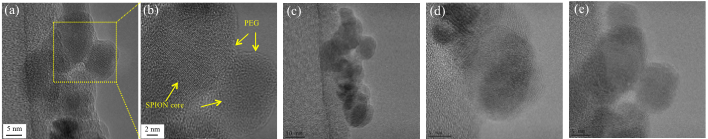


**Figure S2.** (a-e) Bright field HRTEM images of PEG-SPION showing Fe_3_O_4_ cores fully coated with PEG.


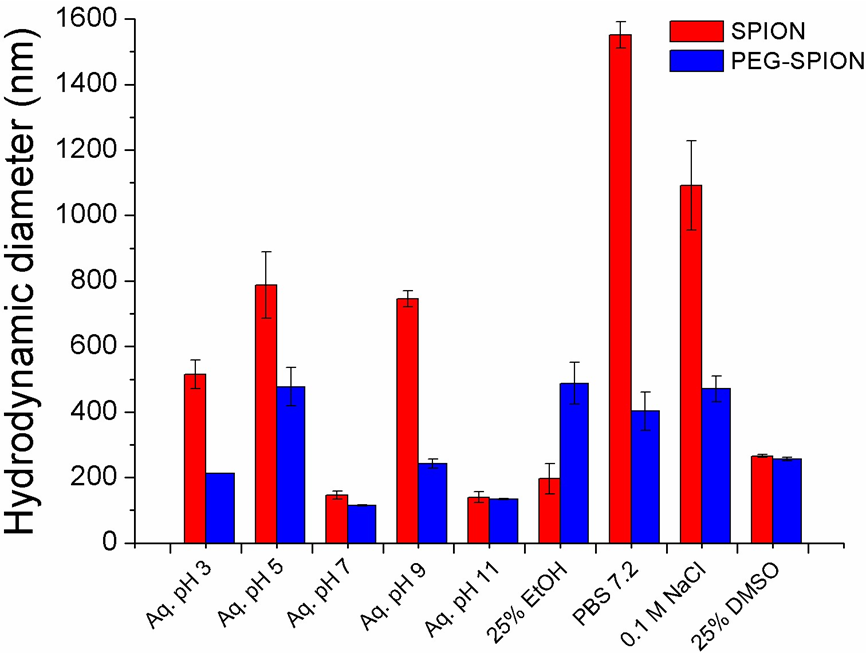


**Figure S3.** Zeta potential measurements showing the isoelectric point (IEP) of SPION and SPION stabilized with PEG (3350).

**
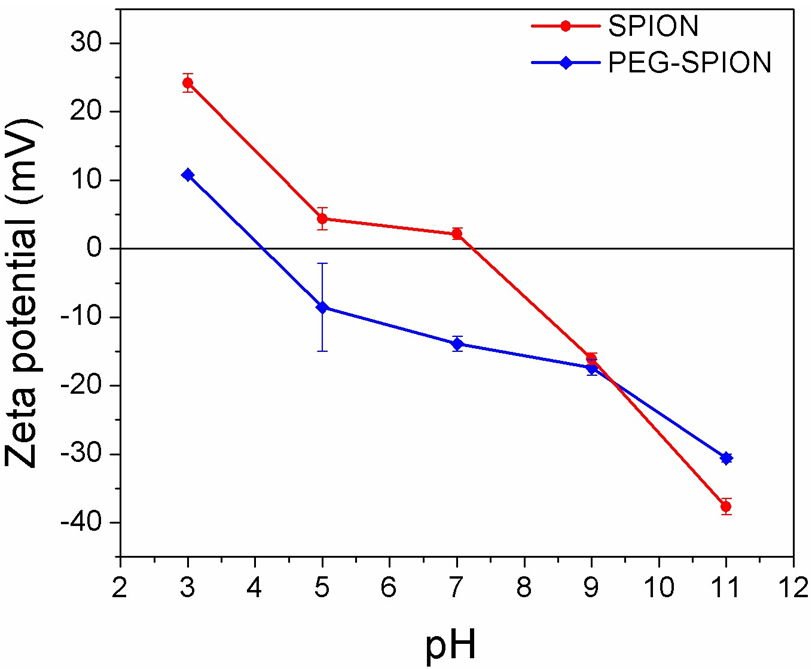
**

**Figure S4.** DLS measurements of SPION and PEG-SPION dispersed in deionized water.


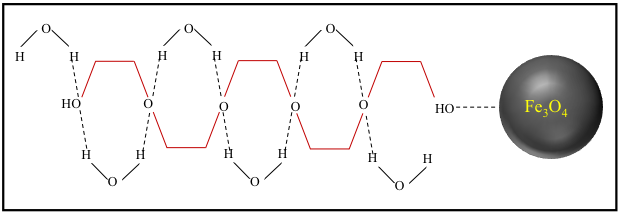


**Figure S5.** A proposed mechanism of dipole cationic binding of ether group of PEG to Fe_3_O_4_ surface and hydration process of PEG for aqueous dispersibility.
